# Draft Genome Sequence of the Decomposition Fungus *Aquanectria penicillioides* Strain NNIBRFG19

**DOI:** 10.1128/MRA.01349-18

**Published:** 2019-01-10

**Authors:** Jaeduk Goh, Hye Yeon Mun, Yoosun Oh, Namil Chung

**Affiliations:** a Fungal Resources Research Division, Nakdonggang National Institute of Biological Resources, Sangju, Republic of Korea; Broad Institute of MIT and Harvard University

## Abstract

Aquanectria penicillioides is a common aquatic fungal species. Here, we report the 53.7-Mb draft genome sequence of *A. penicillioides* strain NNIBRFG19, which has an overall G+C content of 47.93%, comprising 13 scaffolds with an *N*_50_ value of 4.932 Mb.

## ANNOUNCEMENT

Aquanectria penicillioides (basionym: Flagellospora penicillioides, teleomorph: Nectria penicillioides) is a common aquatic fungus and is one of the decomposition fungi of leaf litter in freshwater ecosystems (1, 2). Strain NNIBRFG19 was first reported as A. penicillioides, isolated from plant litter in Samcheok, Republic of Korea (3). Here, we report the first draft genome sequence of this fungus as one of the representative aquatic fungi in a freshwater environment.

A mycelial plug grown on potato dextrose agar (PDA; BD, Franklin Lakes, NJ) at 25°C, was used as the initial inoculum for the liquid culture. Genomic DNA and total RNA were extracted from mycelia grown in potato dextrose broth by shaking at 180 rpm for 1 week at 25°C under dark conditions using the DNeasy minikit (Qiagen, Valencia, CA) and the Easy-spin total RNA extraction kit (iNtRON, Sungnam, Republic of Korea), respectively. The genome sequence of NNIBRFG19 was obtained through a combination of four PacBio RS II single-molecule real-time (SMRT) cells (total 731,038 reads and 6,066,032,880 bp) and one paired-end (total 42,974,130 reads and 4,340,387,130 bp) and two mate pair libraries (total 93,212,966 reads and 9.41 Gb) on the HiSeq 2500 Illumina platform (Theragen Etex Bio, Suwon, Republic of Korea). The PacBio library and a paired-end Illumina library with an insert size of 400 bp were constructed after DNA fragmentation, A-tailing, phosphorylation, and adapter ligation. Two mate pair Illumina libraries with an insert size of 10 kb were constructed with the Nextera mate pair library preparation kit. An RNA-seq library was constructed by paired-end sequencing after fragmentation by random hexamer priming. Reads of RNA-seq were mapped to the assembled genome using TopHat v2 (4). The quality of the reads was checked using FastQC (http://www.bioinformatics.babraham.ac.uk/projects/fastqc). The reads were preprocessed by mHAP in Canu v1.6 (default parameters) for PacBio raw data (5), Trimmomatic v0.30 for paired-end sequencing (default parameters) (6), and NextClip v1.3 for mate pair sequencing (7) (min_length, 46; trim_ends, 0; removal of PCR duplication). The reads were assembled by Canu v1.6 for PacBio reads (default parameters) and SOAPdenovo v2 for Illumina reads (default parameters, k-mer frequency = 65) (8), and the two assemblies were merged by HaploMerger2 v20151124 with default parameters (9).

The draft genome of NNIBRFG19 consisted of 13 contigs with an *N*_50_ value of 4.932 Mb covering 341.72-fold of the genome (the average depth of the genome calculated using all reads). The total length of the assembled genome was 53,763,490 bp with a G+C content of 47.93%. The maximum and minimum scaffold lengths were 8,280,041 bp and 17,759 bp, respectively. A total of 13,658 genes were predicted by AUGUSTUS v3.2.1 using transcript alignment and protein-based alignment among similar species (10). The average length of the predicted genes was 1,604 bp, and the average number of exons was 2.86. This draft genome sequence will support functional genetic research on the mechanism of leaf litter decomposition by aquatic fungi.

### Data availability.

The draft genome sequence of A. penicillioides strain NNIBRFG19 has been deposited in GenBank under accession number PYIU00000000. The SRA accession numbers are SRR8067720, SRR8067732, SRR7800907 and SRR8204250. This paper describes the first version of the genome for this strain.
